# Cognitive functioning in children and adolescents with cerebral palsy: protocol for the Danish CPCog-Youth study

**DOI:** 10.1186/s12887-024-05305-w

**Published:** 2024-12-27

**Authors:** Camilla Funch Uhre, Ida Dyhr Caspersen, Carsten Lose, Gija Rackauskaite, Ro Robotham, Christina E. Hoei-Hansen

**Affiliations:** 1 Center for Clinical Neuropsychology, Children and Adolescents, Rigshospitalet, Blegdamsvej 9, Copenhagen, 2100 Denmark; 2 Center for Rehabilitation of Brain Injury, Copenhagen, Denmark; 3 Department of Clinical Medicine, University of Aarhus, Aarhus, Denmark; 4 Department of Paediatrics and Adolescent Medicine, Aarhus University Hospital, Aarhus, Denmark; 5 Department of Psychology, University of Copenhagen, Copenhagen, Denmark; 6 Department of Paediatrics and Adolescent Medicine, University Hospital Rigshospitalet, Copenhagen, Denmark; 7 Department of Clinical Medicine, University of Copenhagen, Copenhagen, Denmark

**Keywords:** Cerebral palsy, Cognition, Pediatric neuropsychology, Neurocognitive tests

## Abstract

**Background:**

Many children and adolescents with cerebral palsy (CP) experience cognitive difficulties, impacting their academic, social, and emotional well-being. A Danish study from 2023 revealed that merely 40% of individuals with CP complete their elementary school education, and previous neuropsychological studies have found that most children and adolescents with CP experience cognitive difficulties. Yet, cognitive functioning is often assumed rather than assessed, and CP follow-up programs focus predominantly on physical functioning. Recognizing this gap, we aim to investigate cognitive functioning in children and adolescents with CP in mainstream schools in Denmark, using standardized neuropsychological tests. Our objective is to provide valuable insights to guide clinical practice on cognitive assessment and intervention, ultimately fostering a more effective support system for individuals with CP.

**Methods:**

The study employs a cross-sectional design, involving 100 children and adolescents with CP aged 11–15 years attending mainstream schools in Denmark. Cognitive assessment includes the core battery from a proposed Nordic test protocol for children and adolescents with CP – the CP*Cog –* encompassing general cognitive functioning (intelligence), executive functions, and visuo-motor skills. Supplementary tests cover verbal and nonverbal short-term memory, attention, fatigue, and mental health. The assessment protocol will also be evaluated by parents of participating children and adolescents with CP. The project is conducted collaboratively by the neuropsychological teams at three assessment sites, in conjunction with the Center for Cerebral Palsy, Rigshospitalet, Aarhus University Hospital, and Aalborg University Hospital. The study benefits from an external advisory board comprising leading international CP experts.

**Discussion:**

Cognitive difficulties in children and adolescents with CP are often overlooked. Few cognitive studies exist, with some providing only estimated cognitive functioning. The CP*Cog*-Youth study will help advance our understanding of the cognitive challenges experienced by children and adolescents attending mainstream schools. The findings will contribute to the development of targeted cognitive assessment and intervention programs for individuals with CP, addressing this commonly neglected aspect of care. By also seeking feedback from participating families and the involved neuropsychologists, we aim to investigate whether the proposed cognitive assessment is feasible and beneficial in real-world settings.

**Trial registration:**

The study was registered on ClinicalTrials.gov on June 27, 2023 (ID: NCT05921422).

## Background

Cerebral palsy (CP) is a group of motor disorders caused by a disruption of the developing brain during infancy or the prenatal period [1]. While CP is defined by motor impairments, cognitive impairments are common [2, 3]. Cognitive impairments are associated with substantial academic, social, and emotional challenges that often increase with age, as schoolwork and social activities become more demanding [4, 5]. Yet, in most countries, cognitive assessment is not systematically offered to individuals with CP.

Prior studies suggest that many individuals with CP have cognitive difficulties, ranging from mild and delimited to severe and global impairments [3, 6, 7]. Across CP subtypes, intellectual impairments affect about 30% of children with CP [8], often combined with pronounced visuo-perceptual/visuo-constructive difficulties [9–12]. For children with an IQ within the normal range, CP is still associated with a high frequency of specific learning disorders [13], and slowed processing speed [14], as well as impaired sustained attention, inhibitory control, and working memory [15–18]. Moreover, individuals with CP are at high risk of falling out of the education system and work force [19], and show increased symptoms of attention-deficit/hyperactivity disorder (ADHD), anxiety, and conduct disorders [20, 21]. A Danish study from 2023 documented that only 40% of children and adolescents with CP complete their elementary school education, and that children with CP achieve lower grade averages than their peers [22]. These findings raise concerns, particularly considering the very limited access to special education support in mainstream educational settings, where children and adolescents with CP are frequently expected to perform academically and socially on par with their peers without CP.

In the Nordic countries, children and adolescents with CP are commonly assessed in a CP follow-up program [23] with a predominant focus on physical condition and motor functions. While cognitive impairment correlates with the degree of motor impairment at the group level [24], cognitive functioning cannot be inferred based on motor functioning or type of CP at the individual level [3, 25]. Cognitive impairments therefore run the risk of being misjudged or entirely overlooked. Children and adolescents with more discrete cognitive impairments are more likely to have their cognitive challenges overlooked, compared to children and adolescents with marked and global cognitive difficulties that are more obvious to their surroundings, including caregivers and schoolteachers. Whether subtle or substantial, unaddressed cognitive impairments may not only reduce academic achievement, but may also affect mental health and reduce quality of life [26, 27] Nordic CP user organizations therefore recently called for systematic follow-up of cognition, which led to the development of a protocol for cognitive assessment: the CP*Cog*, published in 2016 [5].

The present study aims to thoroughly investigate cognitive functioning and overall well-being of children and adolescents with CP enrolled in mainstream schools in Denmark. Using core tests from the CP*Cog* along with a supplemental test battery, we seek to provide a comprehensive understanding of their cognitive abilities and mental health status, delineating the nature and severity of potential cognitive, psychopathological, social, and emotional difficulties. Tolerability and utility of the assessment protocol will be evaluated by the participating families, as well as the neuropsychologists administering the tests. Ultimately, we aim to provide scientific insights that can translate into actionable guidelines for cognitive assessments and interventions for individuals with CP.

## Methods

### Study design

The CP*Cog*-Youth study is a cross-sectional study with three data-collection sites – two in Copenhagen, Denmark (Center for Rehabilitation of Brain Injury and Center for Clinical Neuropsychology, Children and Adolescents, Rigshospitalet) and one in Aalborg, Denmark (Aalborg University Hospital).

### Participants

We aim to include 100 children and adolescents with CP. See Table 1 for criteria of inclusion and exclusion.

**Table 1:** Eligibility criteria

Inclusion	Exclusion
▪ 11–15 years old. ▪ Diagnosed with CP (irrespective of type and severity). ▪ Attends a Danish mainstream school at time of enrollment.	▪ Does not speak or understand Danish at the level necessary to participate (as evaluated by the research team).

### Assessment battery and outcomes

All participants will be assessed using core tests from the CP*Cog as* well as a comprehensive supplemental battery developed for this study (see Table 2). The core battery comprises standardized tests of general cognitive functioning (intelligence), visual-spatial abilities, and a questionnaire which assesses executive functioning in daily life. The supplemental battery includes standardized tests and questionnaires that assess attention, executive functioning, memory, fatigue, and mental health. Tests and chosen outcomes for analyses are detailed in Table 2.

**Table 2:** Assessment battery

	Test	Type	Outcome
**Domain (CPCog)**			
General cognitive functioning	Wechsler Intelligence Scale for Children – Fifth Edition (WISC-V) [28]	Test	General Ability Index (GAI)
Visual-spatial function	The Beery-Buktenica Developmental Test of Visual-Motor Integration (Beery VMI) [29]	Test	Beery VMI score
Executive functioning	Behavior Rating Inventory of Executive Function – Second Edition (BRIEF-2), parent version [30]	Questionnaire	Cognitive Regulation Index
**Domain (supplementary)**			
Executive functioning	Delis-Kaplan Executive Function System Trail Making Test part A and B (D-KEFS TMT) [31]	Test	Completion time (number sequencing; number-letter switching)
	D-KEFS Verbal Fluency [31]		Number of correct words named (category fluency; category-switching)
Attention	The Test of Variables of Attention 9 (T.O.V.A.) [32]	Test	Response sensitivity score (d’)
	ADHD-rs [33]	Questionnaire	Total scores of inattention, hyperactivity, and behavior scales
Memory	Test of Memory and Learning, Second Edition (TOMAL-2) [34]	Test	Scale scores of selected verbal subtests (Word Selective Reminding; Object Recall) and nonverbal subtests (Abstract Visual Memory; Visual Sequential Memory)
Working memory	Working Memory subtests (WISC-V) [28]	Test	Working Memory Index (WISC-V)
Processing speed	Cancellation (WISC-V) [28]	Test	Scale score of the cancellation test
Fatigue	PedsQL Multidimensional Fatigue Scale, parent and child version [35]	Questionnaire	Total score
Adaptive behavioral functioning	The Adaptive Behavior Assessment System, Third Edition (ABAS-3) [36]	Questionnaire	The General Adaptive Composite (GAC)
Mental Health	Behavior Assessment System for Children, Third Edition (BASC-3), parent and child version [37]	Questionnaire	Index scores for ADHD probability, autism spectrum disorder probability, and emotional behavioral disturbance probability. Clinical scales of anxiety and depression.

### Assessment procedures

The cognitive assessment will take approximately four hours to complete (including breaks) and will be administered in a fixed order over the course of two days (for details see Table 3 below).

The number of assessment days can be adapted if necessary for the individual participant. Adaptations in test procedures will be recorded by the assessor.

**Table 3:** Test session overview

Session	Test	Administration order
Day 1	WISC-V (15 subtests, 90 min)	1
	Beery VMI (10 min)	2
Day 2	D-KEFS Trail Making Task (two subtests, 10 min)	3
	D-KEFS Verbal Fluency (three subtests, 15 min)	4
	TOMAL-2 (four subtests, 25 min)	5
	T.O.V.A (25 min)	6

*Abbreviations* WISC-V = Wechsler Intelligence Scale for Children – Fifth Edition [15]; Beery VMI = The Beery-Buktenica Developmental Test of Visual-Motor Integration (Beery VMI) [16]; D-KEFS = Delis-Kaplan Executive Function System [18]; TOMAL-2 = Test of Memory and Learning, Second Edition [20]; T.O.V.A. = The Test of Variables of Attention 9 [14]

Participants will receive both an oral and a written summary of their assessment results, as well as recommendations for intervention if relevant. When appropriate and needed, we will offer live or virtual meetings to review test results and recommendations with professionals (e.g., teachers and school psychologists) in addition to the individual participants and their families.

The neuropsychologist assessor will be asked to complete a questionnaire regarding the assessment procedure, whether it was doable, or if any tests had to be skipped or adapted to overcome challenges in the test situation. Three months after the assessment, the participant and caregivers will also be invited to complete a survey on their experience of the assessment procedures and the usefulness.

### Data from patient records

We will retrieve the following information from participants medical records and the Danish CP follow-up program (CPOP) [38]: age, sex, CP subtype, CP related tests and treatment, and functional ability classification. Information from patient records will be used to confirm eligibility (i.e., age and CP diagnosis). After inclusion, data on CP subtype and functional ability classification will be used for statistical analyses to test for associations between type and severity of CP and type and severity of cognitive impairment.

### Recruitment

Children and adolescents with CP will be recruited from the neuropediatric departments of the hospitals involved in the Danish CP follow up program: Rigshospitalet, Copenhagen, Denmark, and Aarhus University Hospital, Aarhus, Denmark. Children with CP and their parents will be asked by the pediatrician if they are interested to hear more about the study. Additionally, we will ask the CP user organizations in Denmark (the Elsass Foundation; CP Denmark) and other relevant institutions to assist with recruitment by sharing our study information and contact details. Families that are interested in participation will be contacted by a study doctor or neuropsychologist who will provide oral and written information about the study before inclusion. If the family decides to participate, written consent will be obtained from the legal guardian(s) and from adolescent participants aged 15 or above.

### Statistical analyses

Data from the sites will be compiled and analyzed collectively. Cognitive functioning in participants with CP will be compared to the norms of the included tests and questionnaires. Data will be analyzed using multivariate analysis of variance (MANOVA) and *t*-tests. When relevant and appropriate, the significance level will be adjusted to control for the risk of false positives due to multiple testing (e.g., using Holms-Bonferroni correction). We will conduct within-group analyses to determine the number of children and adolescents with cerebral palsy (CP) who perform significantly below the norm average and assess the number of domains in which these deviations occur. Data collected in the current study will be supplemented by data from the Danish CP registry. Data on CP subtype and functional ability classification will be used for statistical analyses to test for associations between type and severity of CP and type and severity of cognitive impairment. Data from parents (e.g., length of education) will be used to test for correlations between these background factors and the cognitive and mental health outcomes of the included children and adolescents.

Responses on surveys will be explored using descriptive statistics (e.g., proportion that found the assessment and recommendations helpful) and free-text responses will be evaluated using qualitative methods, grouping answers into a set of main points/themes, if possible.

### Power calculation

The CP*Cog*-DK study will be among the largest cognitive studies in children and adolescents with CP. Power calculation is difficult as our study includes outcomes across a broad range of cognitive domains and existing literature on neuropsychological test results for children and adolescents with CP is limited.

### Data management

Raw data will be stored at the site of assessment and in accordance with Danish national regulations. Additionally, data will be entered into an electronic database (REDCap), stored on a secure server hosted by the Capital Region, Denmark.

## Discussion

This study aims to characterize children and adolescents with CP on measures of cognition and mental health. Study participants attend Danish elementary schools in which they are largely expected to perform comparably to their peers without CP. Yet, we know that these children and adolescents face a high risk of not completing their school education [22], highlighting a gap of knowledge as to why this is and how to properly intervene.

While cognitive difficulties are widely acknowledged to affect a significant proportion of children and adolescents with CP, studies applying formal cognitive testing on a large scale are notably lacking. Systematic assessment of both physiological and cognitive functioning in CP is indispensable for developing standardized guidelines that uphold the formal rights of children and adolescents with CP. Moreover, raising a child with special needs can be challenging, and parents of children with CP experience more stress and reduced mental and physical health compared to parents of typically developing children [39, 40]. Parental stress does not depend on the ability-level of the child but is moderated by a variety of factors including psychosocial resources of the family and accessibility of health care services [39]. Access to systematic follow-up that addresses the entire range of parental concerns on the development of the child could be an important way of alleviating caregiver stress in addition to supporting the individual child [39–41].

Even in cases when children with CP do receive cognitive evaluation, they are often faced with tests that rely on a speeded motor-output for interpretation. The Weschler scales, of which WISC-V is the latest version, is a world-wide gold standard test battery for assessing general cognitive functioning (IQ). Norms are based on populations of children and adolescents with normal fine-motor skills and eye-hand coordination. As performance on core subtasks (such as Block Design and Coding) heavily depend on a coordinated visuo-motor output, test validity may be compromised when assessing children with CP, even in the absence of obvious motor impairments [42–45]. To address this issue, the present study aims to evaluate the feasibility of both core and supplementary tasks of the WISC-V in assessing general cognitive functioning of children with CP. Pioneer efforts to test cognitive domains across modalities have been made [46] and should be further explored to guide clinicians on how to properly adapt assessment methods and ensure accurate outcomes when assessing children and adolescents with CP.

In conclusion, by gaining a better understanding of cognitive functioning and well-being, this study will help pave the way for targeted assessment and intervention tailored to the specific needs of children and adolescents with CP.

## Data Availability

No datasets were generated or analysed during the current study.

## Declarations

### Authors’ and study team information

The study will be carried out in collaboration between neuropsychologists and neuropediatricians affiliated with the primary sites of assessment, as well as national and international CP experts. The study team comprises: MSc, PhD, Camilla Funch Uhre, Center for Clinical Neuropsychology, Children and Adolescents, Rigshospitalet, Denmark (PI and sponsor). Assistant Professor Ro J. Robotham, Department of Psychology, University of Copenhagen, Denmark (member of steering committee). Professor, consultant pediatric neurologist, Dr.Med, Christina E. Høi-Hansen, Department of Children and Adolescents, Rigshospitalet and University of Copenhagen, Denmark (member of steering committee). MSc, PhD, Ida Dyhr Caspersen, Center for Clinical Neuropsychology, Children and Adolescents, Rigshospitalet, Denmark (Postdoc, primary assessor at Rigshospitalet). Associate Professor, PhD, consultant pediatric neurologist Gija Rackauskaite, Aarhus University Hospital, Denmark (primary responsible for recruitment at Aarhus University Hospital). MSc Carsten Lose, Head of Department for Children and Adolescents, Center for Rehabilitation of Brain Injury, Denmark (Senior neuropsychologist, member of steering committee, supervisor and responsible for assessments at the Center for Rehabilitation of Brain Injury). MSc Frank Humle, Director of the Center for Rehabilitation of Brain Injury, Denmark. MSc Louise Maria Torp Sørensen, Aalborg University Hospital, Aalborg, Denmark. Associate Professor, PhD, Jonas Kjeldbjerg Hansen, Aalborg University Hospital, Aalborg, Denmark. The study team develops and coordinates the study, including recruitment of participants, data collection, and dissemination of results. In addition to the study team, the project is overseen by an advisory board including: Associate Professor, Kristine Stadskleiv, Department of Special Needs Education, University of Oslo, Norway. Associate Professor Ann Alriksson-Schmidt, Department of Clinical Sciences, Lund University, Sweden.

## Abbreviations

CP: Cerebral Palsy
CPCog: A proposed Nordic test protocol for children and adolescents with CP
CPOP: The Danish CP registry
GP: General practitioner
ADHD: Attention-deficit/Hyperactivity Disorder
WISC-V: Wechsler Intelligence Scale for Children – Fifth Edition
Beery VMI: The Beery-Buktenica Developmental Test of Visual-Motor Integration
D-KEFS: Delis-Kaplan Executive Function System
TOMAL-2: Test of Memory and Learning, Second Edition
T.O.V.A: The Test of Variables of Attention
PedsQL: Pediatric Quality of Life Inventory
